# Retraction Note: γ-Aminobutyric acid (GABA) administration improves action selection processes: a randomised controlled trial

**DOI:** 10.1038/s41598-022-13116-1

**Published:** 2022-05-25

**Authors:** Laura Steenbergen, Roberta Sellaro, Ann-Kathrin Stock, Christian Beste, Lorenza S. Colzato

**Affiliations:** 1 Institute for Psychological Research, Leiden Institute for Brain and Cognition, Leiden University, Leiden, The Netherlands; 2 Cognitive Neurophysiology, Department of Child and Adolescent Psychiatry, Faculty of Medicine of the TU Dresden, Dresden, Germany

Retraction of: *Scientific Reports* 10.1038/srep12770, published online 31 July 2015

The Editors have retracted this article.

An investigation by Universiteit Leiden has concluded^1^ that that data from 16 participants were excluded from the results of the trial reported in this article. The exclusion of these data is not disclosed in the article. The Editors therefore no longer have confidence in the results and conclusions presented.

Laura Steenbergen, Roberta Sellaro, Ann-Kathrin Stock, and Christian Beste agree with this retraction. Lorenza S. Colzato disagrees with this retraction.

## References

[1] Results of Official Investigation at Universiteit Leiden; 20–02. https://www.organisatiegids.universiteitleiden.nl/binaries/content/assets/ul2staff/organisatiegids/universitaire-commissies/cwi/supplementary-decision-cwi-20-02-c2b_en_redacted.pdf (17 May 2022).

